# Wingless Signalling Alters the Levels, Subcellular Distribution and Dynamics of Armadillo and E-Cadherin in Third Instar Larval Wing Imaginal Discs

**DOI:** 10.1371/journal.pone.0002893

**Published:** 2008-08-06

**Authors:** Ildiko M. L. Somorjai, Alfonso Martinez-Arias

**Affiliations:** 1 Department of Genetics, University of Cambridge, Cambridge, United Kingdom; Temasek Life Sciences Laboratory, Singapore

## Abstract

**Background:**

Armadillo, the *Drosophila* orthologue of vertebrate ß-catenin, plays a dual role as the key effector of Wingless/Wnt1 signalling, and as a bridge between E-Cadherin and the actin cytoskeleton. In the absence of ligand, Armadillo is phosphorylated and targeted to the proteasome. Upon binding of Wg to its receptors, the “degradation complex” is inhibited; Armadillo is stabilised and enters the nucleus to transcribe targets.

**Methodology/Principal Findings:**

Although the relationship between signalling and adhesion has been extensively studied, few *in vivo* data exist concerning how the “transcriptional” and “adhesive” pools of Armadillo are regulated to orchestrate development. We have therefore addressed how the subcellular distribution of Armadillo and its association with E-Cadherin change in larval wing imaginal discs, under wild type conditions and upon signalling. Using confocal microscopy, we show that Armadillo and E-Cadherin are spatio-temporally regulated during development, and that a punctate species becomes concentrated in a subapical compartment in response to Wingless. In order to further dissect this phenomenon, we overexpressed Armadillo mutants exhibiting different levels of activity and stability, but retaining E-Cadherin binding. Arm^S10^ displaces endogenous Armadillo from the AJ and the basolateral membrane, while leaving E-Cadherin relatively undisturbed. Surprisingly, ΔNArm^1–155^ caused displacement of both Armadillo and E-Cadherin, results supported by our novel method of quantification. However, only membrane-targeted Myr-ΔNArm^1–155^ produced comparable nuclear accumulation of Armadillo and signalling to Arm^S10^. These experiments also highlighted a row of cells at the A/P boundary depleted of E-Cadherin at the AJ, but containing actin.

**Conclusions/Significance:**

Taken together, our results provide *in vivo* evidence for a complex non-linear relationship between Armadillo levels, subcellular distribution and Wingless signalling. Moreover, this study highlights the importance of Armadillo in regulating the subcellular distribution of E-Cadherin

## Introduction

During development, morphogenetic movements require the coordinated input of signal transduction and physical restructuring to produce fields of cells with appropriate spatio-temporal patterning and fate. One efficient means by which to achieve this is to recycle the cellular machinery for multiple functions, for example by directly linking signalling to adhesion. *Drosophila* Armadillo, and by extension its vertebrate orthologue β-catenin, is perhaps one of the best candidates for such a molecular integrator. In effect, Armadillo/β-catenin plays dual roles in transducing Wingless/Wnt, and in bridging E-Cadherin to the actin cytoskeleton (reviewed in [1]).

Wingless (Wg), the *Drosophila* orthologue of Wnt-1, is a diffusible glycoprotein with concentration-dependent effects, and is critical to patterning and growth of the embryo and larval imaginal epithelium [2], [3]. The essence of canonical Wg signalling is the modulation of the amount and activity of Armadillo. There are two pools of Armadillo, one at the adherens junction and another deemed to be cytoplasmic. In the absence of Wg, the cytoplasmic pool is low due to a steady degradation of Armadillo, mediated by a complex centered around the protein Axin. In this complex, phosphorylation of Armadillo by Shaggy/GSK3β kinase targets it for proteasomal degradation. Upon Wg signalling, a receptor complex containing Frizzled (Fz) and Arrow/LRP6, through the action of the adaptor protein Dishevelled, leads to the recruitment of the Axin complex to the membrane, where it is dismantled and destroyed [4]–[8]. As a result of these interactions, the cytoplasmic levels of Armadillo rise and it can enter the nucleus, where it interacts with LEF1/TCF to modulate transcription of Wg target genes [3], [9].

A different pool of Armadillo is bound to E-Cadherin at the adherens junction (AJ), a specialised structure in the subapical lateral membrane linking epithelial cells [10]. E-Cadherin is a homophilic adhesion molecule whose extracellular EGF repeats, bound to Ca^2+^, promote epithelial integrity [11]. Quantitative changes in E-Cadherin contribute to cell sorting [12], [13], and misexpressed E-Cadherin is responsible for invasive behaviour and cellular transformation in some cancers, through disregulation of β-catenin [14], [15]. E-Cadherin's primary role in cellular rearrangements is accomplished via its link, through Armadillo, to α-catenin and a battery of other adaptor proteins, and hence to the actin cytoskeleton [11]. In vertebrates, a paralogue of β-catenin, Plakoglobin, fulfills the adhesive function at the AJ, but both are able to interact with E-Cadherin [16]–[18].

Biochemical and structural studies, mostly in vertebrates, have suggested that β-catenin fulfils either its transduction or its adhesive role, but not both simultaneously. The phosphorylation status of β-catenin at key residues is critical to its choice of binding partner [19]–[21], and the N- and C-termini, which act as Wnt transactivation domains [22]–[23], also interact sterically with the central Armadillo repeats. This regulates the “open” or “closed” conformations of β-catenin, thereby affecting its ability to bind to E-Cadherin or TCF [24]–[25]. Thus, these studies have provided a mechanism by which β-catenin might “choose” between signalling and adhesion states, through competition and affinity of binding partners, including itself [26]–[27].

At center stage is the question of how Armadillo/β-catenin mediates its Wg/Wnt signalling function *in vivo*. However, such studies have been difficult both to undertake and to interpret, precisely because of the protein's vital role in the integrity of AJs. This issue has been addressed, in embryos for the most part, by overexpressing various mutants in hypomorphic backgrounds [28]–[30], with the consensus that much of their activity is mediated by the endogenous protein [31]–[34]. Studies have also pointed to the important correlation between stability, rather than levels, and signalling activity of β-catenin [35]. Data also suggest that control of β-catenin nuclear import/export and compartmental retention represent an additional level of regulation [36]–[39], whose effect may be to mask the relative contributions of levels *vs* activity in mediating signalling.

Critical to our understanding of Wg/Wnt signalling is how the E-Cadherin-associated and signalling pools of Armadillo/β-catenin communicate. It has been demonstrated that, concomitant with the stabilisation and increase in cytoplasmic levels of Armadillo/β-catenin induced by Wg/Wnt signalling, there is a reduction in E-Cadherin-bound protein at the membrane [40]. Conversely, overexpression of E-Cadherin is correlated with reduction in β-catenin levels and signalling, resulting presumably from sequestration of free protein [14], [40], [41]. The observations that both E-Cadherin exocytosis to the basolateral membrane, and efficient exit from the ER require Armadillo/β-catenin [42], [43] further hint at an important interaction between E-Cadherin and Armadillo in movement between different subcellular compartments.

Few data exist in animal models on the subcellular distribution of Armadillo/β-catenin and E-Cadherin in the context of their function, with notable exceptions [28], [35], [44]–[48]. What emerges is a picture of a complex relationship between β-catenin's distribution, activity and signalling potential, mediated in part by its association with E-Cadherin. Further, these results provide an interesting *in vivo* counterpart to recent biochemical evidence suggesting that α-catenin can bind β-catenin or actin, but not both [49], [50]. These studies suggest that conventional models in which β-catenin is either found in complex with E-Cadherin and α-catenin to mediate actin dynamics, or is free to mediate its signalling functions, are overly simplistic.

In an effort to help elucidate the relationship between the different pools of β-catenin and Wingless signalling, we have undertaken a detailed analysis of the subcellular distribution of Armadillo in the wing imaginal discs of *Drosophila*. We show that Armadillo is dynamically regulated during the third larval instar, closely paralleled by E-Cadherin, throughout the apicobasal section of the epithelium. Most importantly, we observe that in response to Wingless signalling, Armadillo is found in a subapical punctate distribution. We have also analyzed the consequences of expressing Armadillo mutants for the distribution of endogenous Armadillo and E-Cadherin. We find a clear dissociation between endogenous Armadillo and E-Cadherin both at the membrane and in the AJ, made evident by the use of our novel method of quantification of subcellular levels. Our results provide new insight into the dynamics of Armadillo/E-Cadherin *in vivo*, and suggest that competition between mutants and endogenous Armadillo may affect recycling of E-Cadherin to the membrane and the AJ. In addition, adult phenotypes may hint at changes in the relationship between AJ components and the cytoskeleton, particularly at the A/P boundary.

## Methods

### Fly work

**Figure 1:**
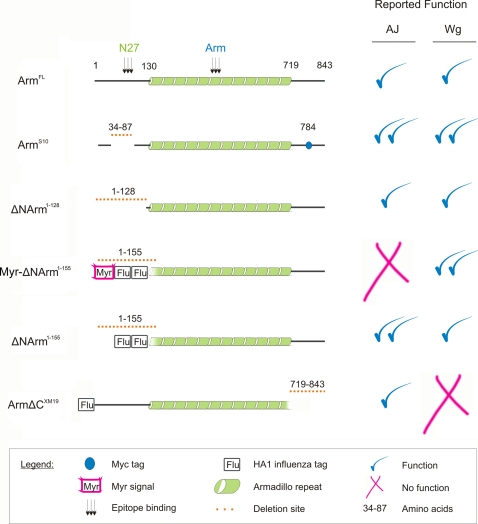
Armadillo UAS constructs used and their proposed function in AJs and Wingless signalling. Arm^FL^ is a full length protein containing N-terminal, Armadillo repeat and C-terminal domains of the wild type protein, and functions in both adherens junctions (AJ) and in Wingless (Wg) signalling. The epitope binding sites of the two antibodies used in this study are shown in the N-terminal (N27) and Armadillo (Arm) repeats. The Arm^S10^ mutant contains the same Myc tag as well as a deletion of amino acids 37–84 in the N-terminus, including the Sgg/GSK3β phosphorylation site, and is a constitutive form activating Wg targets. It is also very stable in the AJ. Three Armadillo constructs lack almost the entire N-terminus and include ΔNArm^1–128^ (amino acids 1–128 deleted), Myr-ΔNArm^1–155^ and ΔNArm^1–155^ (both lacking amino acids 1–155). While ΔNArm^1–128^ is untagged and recapitulates wild type AJ and Wg function, Myr-ΔNArm^1–155^ and ΔNArm^1–155^ both possess tags derived from the influenza virus haemagglutinin protein HA1 (Flu) within the deleted portion of the N-terminus,and act as highly activated forms. Myr-ΔNArm^1–155^ also contains a myristylation (Myr) signal sequence to target it to membranes. Finally, the ArmΔC^XM19^ form lacks the entire C-terminus, and is Flu tagged to allow detection. It is unable to rescue Wg function but like the other constructs has some function in the AJ. Please see text for details.

Fly stocks were maintained at 18°C or 25°C on standard media (10% cornmeal, 9% glucose, 4% yeast, 1% agar and 0.3% nipagin in ethanol), supplemented with yeast. Ectopic expression was achieved with the *GAL4* UAS system [51]. Virgin females carrying the *GAL4* driver were crossed to males carrying UAS constructs to produce progeny with temporally and spatially-restricted patterns of overexpression. *dppGAL4* was used to drive expression in the *decapentaplegic* domain, along a stripe in the anterior compartment at the A/P boundary. Ub-Cadherin-GFP containing stocks simultaneously express GFP-tagged E-Cadherin under a ubiquitous promoter (a gift courtesy of B. Sanson). Similarly, *arm*>armGFP stocks express GFP-tagged Armadillo under the Armadillo promoter [52]. The UAS constructs overexpressed in this study include Wg^E1^, a wild type allele of Wingless [53]; as well a variety of previously described Armadillo mutants termed here Arm^S10^ [30], ArmΔC^XM19^ [34], ΔNArm^1–128^ [30] (amino acids 1–128 deleted), ΔNArm^1–155^ (a gift from G. Struhl; amino acids 1–155 deleted), and Myr-ΔNArm^1–155^ [29] (amino acids 1–155 deleted). The characteristics of the Armadillo constructs, including their proposed function in Wingless signalling and adhesion, are summarised in Figure 1.

### Immunohistochemistry

Wing imaginal discs were dissected from wandering second and third instar larvae, and stained using standard protocols [54], with a few modifications. Immediately upon dissection, each anterior portion of larva was placed in mesh baskets floating in 1× BBS+CaCl_2_ on ice. As many larvae were dissected as possible in the space of 30 minutes, at which point the baskets were transferred to wells containing 4% PFA (paraformaldehyde in BBS+0.1 M CaCl_2_) for fixation. This is critical, as Armadillo protein is quickly degraded (personal observation). Wing discs were mounted in Vectashield (Vector Laboratories) on a slide, and kept at 4°C in the dark prior to confocal microscopy.

Primary antibodies used in this study included α-Armadillo “Arm” (Rabbit; 1∶1000; a gift from H. Müller); α-Armadillo “N27” (N27A1; Mouse; 1∶30; Developmental Studies Hybridoma Bank); α-E-Cadherin “Cad2” (Rat; 1∶20; DSHB); α-Scribble “Scrib” (Rabbit; 1∶2000; a gift from N. Gorfinkiel); α-FasciclinIII “FasIII” (Mouse; 1∶50; DSHB); α-Wingless “4D4” (Mouse; 1∶200; DSHB) and α-Wingless “Wg” (Rabbit; 1∶200; a gift from S. Cumberledge); α-HA “Flu”(Flu); α-Neurexin IV (Rabbit; 1∶1000; a gift from N. Gorfinkiel).

The following fluorescently-labelled secondary antibodies (Molecular Probes) were used at 1∶200 dilution: Alexa Fluor 488 goat anti-mouse, Alexa Fluor 568 goat anti-mouse, Alexa Fluor 488 goat anti-rabbit, Alexa Fluor 568 goat anti-rabbit, Alexa Fluor 547 goat anti-rabbit, Alexa Fluor 488 goat anti-rat, Alexa Fluor 568 goat anti-rat, Alexa Fluor 547 goat anti-rat, Alexa Fluor 568 donkey anti-sheep. In addition, Cy-5 conjugated donkey anti-guinea pig and anti-mouse were used (Jackson Immunoresearch).

For actin visualisation, larvae were fixed as above with the addition of 1 U/ml phalloidin, stained and incubated with Texas-Red-conjugated-phalloidin along with secondary antibodies (Molecular Probes).

### Image acquisition

Images of adult wings were photographed using a Zeiss Axiophot microscope mounted with a camera. Objectives used included 5× and 10× magnification. Wings were oriented with veins L3 and L4 parallel to the horizon; images were subsequently rotated using Photoshop software (version 6.0) to ensure anterior pointed upwards, and the distal wing tip to the left.

All image data on larval wing discs were collected using a Nikon E800 upright microscope with Bio-Rad laser. Objectives used included 10×, 20× (oil immersion) and 60× (oil immersion) magnification, in addition to a further optical zoom of 2×. The iris aperture was set to 3.0 for all image acquisition. Images in red, green and blue (infra-red) channels were taken sequentially to avoid the phenomenon of “bleed-through” across channels. Sections of the same wing disc were taken at 0.5 µm intervals from the AJ, which was considered 0% (see Results). The total number of sections taken between the AJ (0%) and the basal-most surface of the epithelium (100%), being dependent upon the age, size and compression of the wing disc, was therefore variable and only percentages shown.

Confocal images were processed and set into image panels using Photoshop software (version 6.0). Images are shown with anterior pointing left and dorsal upwards, with the wing pouch centred unless otherwise indicated. All confocal images are sub-apical sections (ca. 0–10%) at 60× magnification unless otherwise indicated, with white crosshairs placed at the intersection of A/P and D/V axes. “Calipers” or “dimension bars” are drawn on both confocal and adult wing images to show the extent of signalling domains, and to highlight differences between experimental conditions (i.e. changes in the width of the *patched* expression domain between veins L3 and L4). In all cases, only representative sections and images are shown for clarity.

### Quantification of levels

Using NIH ImageJ software, we undertook to quantify levels of overexpressed mutants, endogenous Armadillo and E-Cadherin within the *dppGAL4* domain of Armadillo construct overexpression. This was initially accomplished at the level of the AJ, and across a field of cells termed the “cellular” compartment, which consists of the basolateral membrane, the cytoplasm, and for the most part nucleus. This compartment was further divided into easily quantifiable nuclear and the basolateral membrane components for all Armadillo constructs (see below).

#### Methodology: “Junctional” and “Cellular” compartments

**Figure 2:**
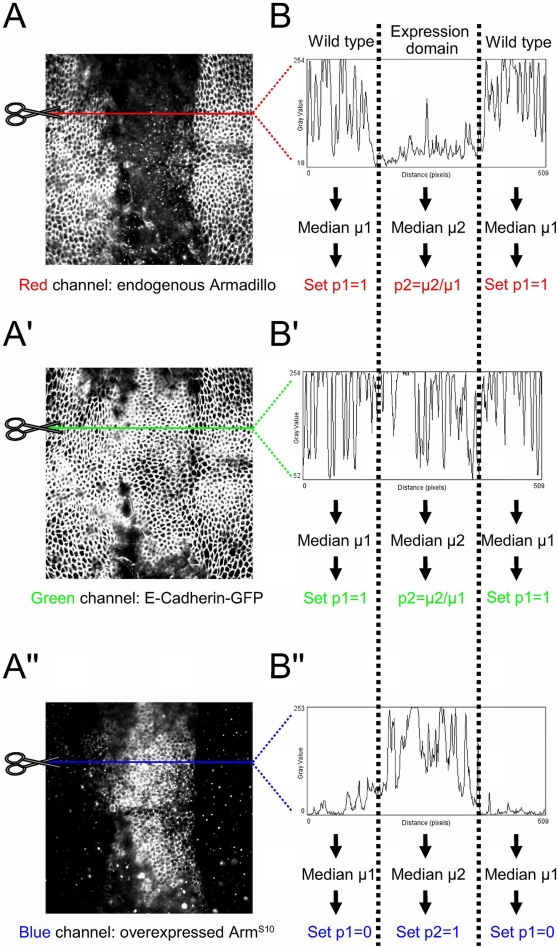
Example illustrating the quantification method developed to compare changes in Armadillo protein levels at the level of the AJ across experiments. (A, A′, A″) UAS Arm^S10^ is overexpressed in the *dppGAL4* domain, which drives expression in a stripe at the A/P boundary. Red, green, and blue channels; representing (A) endogenous Armadillo, (A′) E-Cadherin-GFP under a ubiquitous promoter, and (A″) Arm^S10^, respectively; are assessed separately from the same confocal section, here at the level of the AJ (60×2 magnification). The coloured lines through the images represent the cross-section at which intensity levels were measured. (B, B′, B″) Using NIH ImageJ software, a histogram is produced in which pixel intensity for each pixel is calculated across the confocal section for each channel. Median values are calculated from both wild type tissue (μ1) and the expression domains (μ2). (B, B′) μ1 is used as the baseline value for endogenous protein levels, and is used to set the proportion of protein in the AJ at p1 = 1. The proportion p2 of junctional protein in the expression domain is then calculated as the median value μ2/μ1 and is a fraction of p1. (B″) p1 is set to 0 as no protein is expected outside of the expression domain, while p2 is set to 1 as it is assumed that the maximal amount of Arm^S10^ will reside in the junction within the expression domain. This allows a distinction between zones of high and low expression levels, the latter being a fraction of p2, such that changes in endogenous protein levels can be monitored (not shown).

To illustrate the method, an example of the quantification system used at the level of the AJ in a wing disc overexpressing UAS Arm^S10^ is presented in Figure 2. Confocal images were separated into the component red, green and blue channels (Figure 2A, A′ and A″). A longitudinal section across the *dppGAL4* domain was chosen, and pixel intensity (gray value) plotted against the distance along the X-axis (pixels). Thus, for each channel it was possible to generate a profile plot in which endogenous Armadillo, E-Cadherin and construct levels were assessed (Figure 2B, B′ and B″). In the case of Armadillo and E-Cadherin, levels in the “wild type” cells outside the domain of expression were used as controls with which to compare changes in levels within the *dppGAL4* expression stripe. The expression domain was also separated into “central” and “lateral” domains representing high and low level *dpp* expression, respectively (not shown). The median intensity value for “wild type” and “expression domain” was then calculated for endogenous Armadillo, E-Cadherin and the construct. Median pixel intensity values were used to represent protein levels instead of mean values as the latter are affected by both outliers and departure from normality.

The calculation of proportions or percentages allows comparison across non-homogeneous datasets, and was executed as follows. In the case of “wild type” cells, both endogenous Armadillo and E-Cadherin median values are assumed to represent maximal protein levels; the proportion is set to p1 = 1 (Figure 2B, B′). Conversely, as the construct is not present in wild type cells, the median intensity value should approach 0, and the value is set to p1 = 0 (Figure 2B″).

Within the expression domain, endogenous Armadillo and E-Cadherin levels are expected to be a fraction of those in the flanking “wild type” cells. Therefore, the proportion p2 was calculated as median μ2/μ1 (Figure 2B, B′). For the constructs, p2 is set to equal 1 in the central domain of Arm^S10^ construct expression, where the maximal values of protein levels are expected. This is used to express relative levels of the construct in the lateral domain of construct expression, which will be a fraction of the maximal value.

The same methodology was used to calculate “cellular” protein levels (see below), at a level at least 10%–50% below the AJ. In all cases at least three sections or wing discs were quantified, and the median set of values used.

#### Methodology: “Basolateral” and “nuclear” compartments

**Figure 3:**
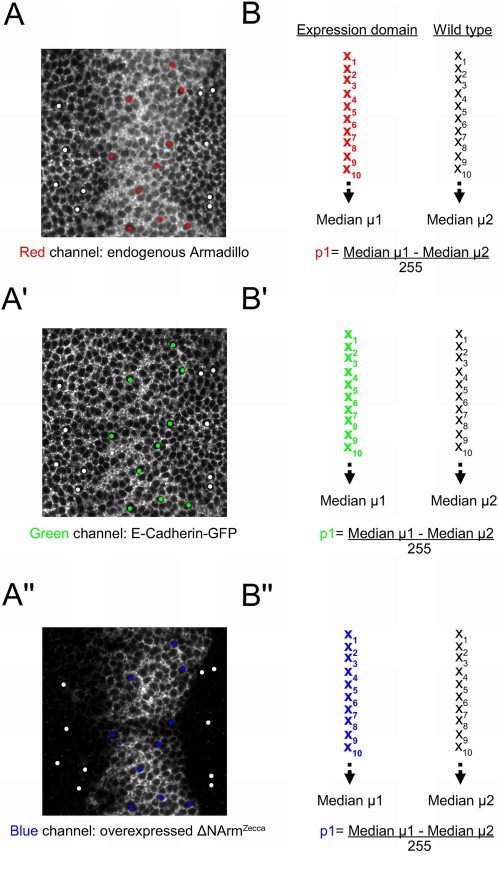
Example illustrating the quantification method developed to compare changes in Armadillo protein levels in the nucleus across experiments. (A, A′, A″) UAS Myr-ΔNArm^1–155^ is overexpressed in the *dppGAL4* domain, which drives expression in a stripe at the A/P boundary. Red, green, and blue channels; representing (A) endogenous Armadillo, (A′) E-Cadherin-GFP under a ubiquitous promoter, and (A″) Myr-ΔNArm^1–155^, respectively; are assessed separately from the same confocal section, here through the cytoplasm approximately 10% below the AJ (60×2 magnification). The red, green and blue spots represent the 10 data points selected from which to calculate median levels within the domain of expression (B, B′, B″). The white spots highlight the data points outside the domain of expression used to remove “background noise”, as the nuclei are expected to have zero pixel intensity here. Thus for each channel, p1 is calculated as μ2 subtracted from μ1, and normalised to a maximal pixel intensity of 255.

Since the cytoplasm is difficult to distinguish from the basolateral membrane in wing discs, and the section will include a large fraction composed of the nucleus, an additional method was used to help clarify changes in levels and subcellular location of proteins (Figure 3). For both the basolateral membrane and the nucleus, 10 points were chosen randomly both in flanking wild type cells and in the central or lateral parts of the expression domain. As with the AJ, a ratio of median pixel intensity values of expression domain over wild type cells was used to quantify changes in Armadillo and E-Cadherin levels. In the case of the construct, “background values” from the wild type domain were subtracted from levels in the expression domain to remove noise from the dataset. The relative intensity of the construct in the lateral and central domains can then be compared. As E-Cadherin does not enter the nucleus (not shown), it was omitted from the nuclear analysis. When levels calculated for the “basolateral” and “nuclear” fractions deviate appreciably from the “cellular” component, it may be possible to infer changes in the cytoplasmic levels of proteins (see results).

## Results

### Dynamic regulation of Armadillo through larval imaginal wing disc development highlights a subapical punctate expression domain

**Figure 4:**
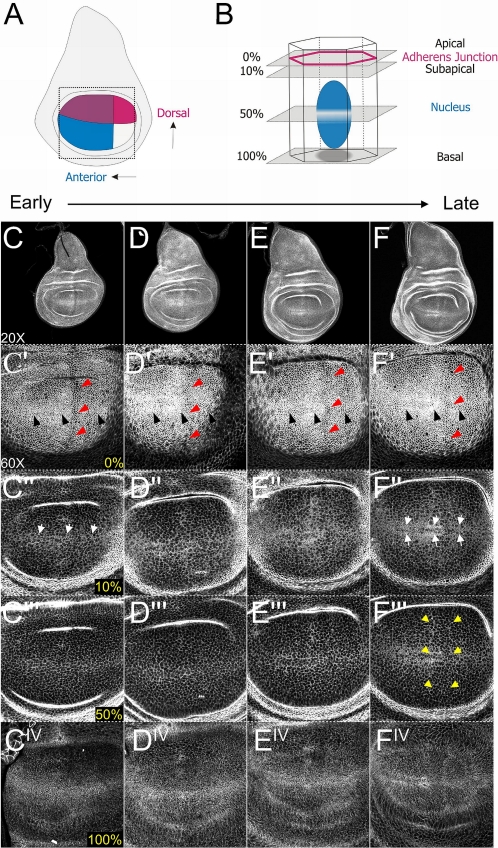
Distribution of Armadillo through 3^rd^ instar larval wing disc development. (A) Diagrammatic representation of a third instar larval wing disc, highlighting the compartments of the wing pouch formed by intersecting Anterior/Posterior (A/P) and Dorsal/Ventral (D/V) boundaries. The dashed box outlines the confocal view at 60× magnification. (B) Diagrammatic representation of apicobasal confocal sections through a single cell of the epithelium, with AJs in red and the nucleus shown in blue. The AJ is considered to be the 0% baseline, with subapical (top 10%), midcellular (50%) and basal (100%) reference points shown. (C–F) The subcellular distribution of endogenous Armadillo changes throughout the development of the 3^rd^ instar larval wing disc as assessed by Armadillo-GFP under the Armadillo promoter [52]. Panels C to F show subsequently older wing discs at 20× magnification. Panels C′ to F^IV^ show changes in subcellular localisation of Armadillo in discs of similar age to C–F at 60× magnification. (C′–F′) Cells at the level of the AJ (0%). Note the distribution of cells along the A/P boundary (red arrowheads), forming a “line” of cells. A single row of cells in early 3^rd^ instar (C′) becomes a series of aligned cells along the D/V boundary by the late 3^rd^ instar in response to Wg and Notch signalling (F′ black arrowheads). (C″–F″) Within the top 10% of the cell, Armadillo has a punctate distribution within the domain of Wg signalling (C″) that resolves into a tramtrack pattern around the D/V domain of expression (F″, white arrows). (C′″–F′″) In addition, at approximately 50% of cell height Armadillo puncta are also stabilised in two vertical stripes along either side of the Hh signalling domain (F′″ yellow arrowheads), most visibly dorsally. These patterns are evident at the basalmost point in the cell as well (C^IV^–F^IV^) The antibody staining with N27A1 recapitulates that of the Armadillo-GFP (not shown).

In an effort to monitor changes in the subcellular localisation of Armadillo during third instar larval wing imaginal disc development, we used a stock expressing an Armadillo-GFP construct under the Armadillo promoter. We focused our analysis on the wing pouch (boxed area, Figure 4A), which everts during pupariation to form the wing blade. A basic coordinate system of intersecting Antero-Posterior (A/P) and Dorso-Ventral (D/V) axes can be defined which is evident upon inspection of Armadillo distribution (see below).

In order to characterise the subcellular distribution of Armadillo, we initially made confocal sections through the entire epithelium of wing imaginal discs at different time points. Since it became evident that the distribution of Armadillo was subject to spatial and temporal changes, we defined apicobasal levels within the imaginal epithelium as percentages of the total height of the epithelium, and focused our analysis on specific levels. Four representative domains of interest were thus identified: 0% at the level of the AJ; 10% comprising a subapical domain, 50% through the nuclei, and 100% at the most basal point in the cells (Figure 4B). We observe changes in the distribution of Armadillo at different levels along the apicobasal axis, from early to late third instar (Figure 4C–F).

**Figure 5:**
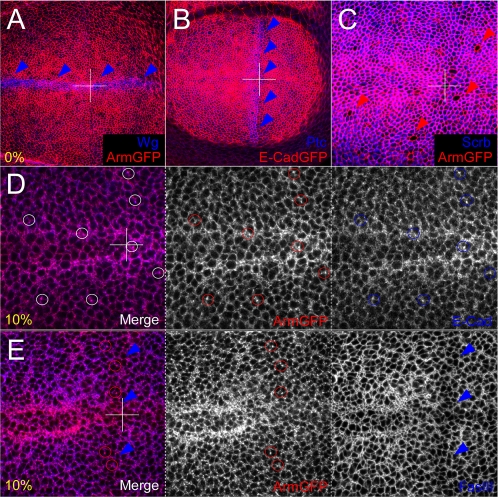
Distribution of Armadillo-GFP relative to basolateral markers. Armadillo-GFP (ArmGFP) and E-CadherinGFP (E-CadGFP) are expressed under endogenous Armadillo ubiquitous promoters, respectively. (A–C) Distribution of ArmGFP (A) and E-CadGFP (B) at AJs (red channels) are coincident; Wingless (Wg) and Patched (Ptc) outline the D/V and A/P boundaries, respectively, which are characterised by aligned cells (arrowheads, blue channel). (C) Mitotic cells express ArmGFP at the AJs, but lack septate junction markers immediately basally (red arrows, Scribble Scrb shown). (D) E-Cadherin (E-Cad, blue) and ArmGFP colocalise at membranes and in puncta (red, blue and white oultines). (E) Fasciclin III (FasIII, blue) crisply and exclusively marks basolateral membranes, with which ArmGFP puncta are closely associated (compare red and blue channels). The downregulation of FasIII at the A/P boundary (blue arrowheads) clearly outlines the ArmGFP puncta there (outlined in red).

At 0%, Armadillo clearly outlines the AJs, showing the arrangement of the imaginal cells with respect to the D/V and A/P axes from early third instar (Figure 4C′). This alignment of cells at the boundaries corresponds to domains of Wingless and Hedgehog signalling (Figure 5A and B), though by late third instar, the increase in cell number makes it difficult to perceive the delineation of cells along the A/P axis. However, by this stage high levels of Armadillo can be observed in two stripes at the D/V boundary (Figure 4F′) on either side of the Wingless expression domain (not shown).

The subapical section (10%) shows an accumulation of Armadillo in puncta correlated with Wingless signalling and which changes from a single stripe along the D/V boundary at the beginning of the third instar to a double stripe by late third instar (Figure 4C″–F″). Interestingly, some accumulation anterior to the A/P boundary was also in evidence early on (Figure 4E″), which by late third instar began to resemble two stripes along the A/P axis dorsally (Figure 4F″). These changes in subcellular distribution were also clearly apparent at 50% (Figure 4C′″–F′″) and 100% (Figure 4C^IV^–F^IV^), with two A/P stripes extending across the wing disc by late third instar (Figure 4F^IV^). For the purposes of discussion, these will be referred to hereafter as the anterior and posterior A/P stripes.

In an effort to characterise further the distribution of Armadillo puncta, we mapped them with reference to various markers of apicobasal polarity. These comprised Crumbs, an apical marker; E-Cadherin, an AJ marker known to associate with Armadillo; and a variety of septate junction and basolateral membrane markers including Fasciclin III, Scribble, Discs Large and Neurexin [79], [80].

At the level of the AJ, unsurprisingly E-Cadherin and Armadillo were entirely coincident (0%, Figure 5A, B). The other markers were either weakly expressed or entirely absent (not shown). The alignment of cells at D/V and A/P boundaries, highlighted by Wingless and Patched expression respectively, was clearly evident at the level of the AJs (Figure 5A, B, blue arrows). In mitotic cells, which can be identified by their larger size, roundedness, and rosette-like appearance, Armadillo was expressed while Fasciclin III, Scribble, Neurexin and Discs Large were absent, suggesting that septate junctions might be dismantled during cell division (Scribble shown, Figure 5C).

Subapically (10% shown), Armadillo and E-Cadherin were strongly associated both in the membrane and in puncta (Figure 5D). However, none of the Armadillo puncta were seen to associate with the other markers (not shown). Fasciclin III, which appears as an exclusive and true membrane marker (Figure 5D, [79]–[80]), highlighted the existence of a membrane-associated but distinct population of Armadillo that localised in puncta. These puncta, while present throughout the wing imaginal disc basal to the AJ (Figure 4C″–F^IV^ and not shown), were however most clearly evident in the subapical domain. With perhaps the exception of a slight increase in subapical Armadillo in Wingless-receiving cells, punctate and membrane Armadillo and E-Cadherin distributions were entirely coincident in wild type wing discs.

Taken together, these observations indicate the existence of spatial and temporal differences in the subcellular distribution of Armadillo during the third larval instar. Furthermore, these changes follow the expression of Wingless protein. Finally, Armadillo and E-Cadherin are tightly associated not only in the AJs, but in membranes and puncta subapically.

### Signalling modulates Armadillo puncta subcellular distribution

The observation that Armadillo puncta were found to be most prevalent in a subapical domain around the D/V boundary where Wingless is expressed (*cf*. Figure 5A), and/or associated with E-Cadherin puncta (Figure 5D), was intriguing. Several hypotheses, which are not mutually exclusive, could be advanced to explain this. In one scenario, Armadillo puncta represent part of the signalling pool that is stabilised by Wingless; in the absence of signalling Armadillo is “safe” in complex with E-Cadherin at the membrane. Alternatively, Wingless does not change the association between Armadillo and E-Cadherin but rather alters the rate of shuttling of Armadillo-E-Cadherin complexes between different compartments, such that Armadillo becomes available for signalling. The idea that E-Cadherin acts to sequester signalling ß-catenin has been proposed (reviewed by [1]), but an in-depth analysis of subcellular changes in ß-catenin is still lacking. We therefore decided to compare the subcellular distribution of Armadillo with that of E-Cadherin and other apicobasal polarity markers upon signalling, by overexpressing a wild type allele of Wingless in the *dpp* domain.

**Figure 6:**
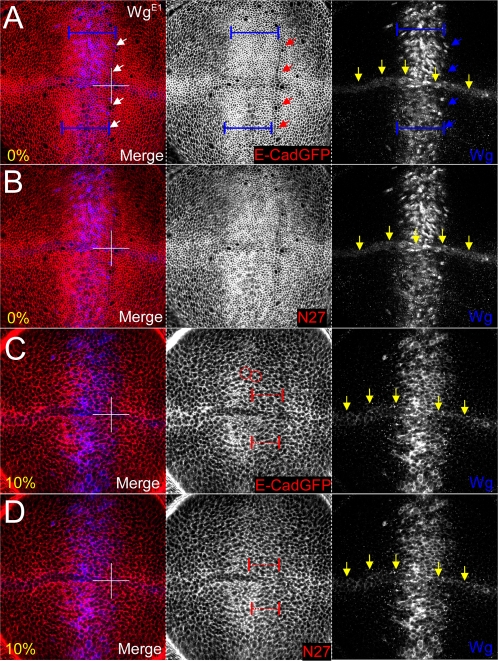
Expression (0% and 10%) of apicobasal polarity markers upon overexpression of UASWg^E1^ under *dppGAL4*. (A, B) At 0%, Wingless signalling changes neither the levels nor the subcellular distribution of apicobasal polarity markers, including E-Cadherin and Armadillo, apically where they are normally situated. The endogenous Wingless (blue channel) at the D/V boundary is indicated by yellow arrows. Both E-Cadherin-GFP expressed ubiquitously (A, E-CadGFP, red channel) and endogenous Armadillo (B, N27, red channel) are stable in the AJ in spite of very high levels of overexpressed Wingless. The A/P boundary is clearly demarcated by aligned cells (A, white, red or blue arrowheads), and the cells seem more densely packed or apically constricted within the overexpression domain (yellow dimension bars). (C, D) At 10%, Wingless signalling induces accumulation of Armadillo puncta subapically, corresponding to a change in E-cadherin levels. The endogenous Wingless (blue) channel at the D/V boundary is indicated by yellow arrows. (C) and (D) represent the same wing disc at the same basal position to allow comparison of protein localisation. (C) E-Cadherin accumulates in puncta (red circles) but also appears to be depleted from the basolateral membranes (yellow arrows). (D) In contrast, Armadillo accumulates in many puncta, of which many correspond to E-Cadherin dots, but does not appear depleted from the basolateral membrane (compare expression in domain delineated by the yellow dimension bars in A and B).

Overexpression of Wingless did not cause appreciable changes in the distribution of apicobasal polarity markers (not shown). However, we observed some changes in the distribution of Armadillo and E-Cadherin that correlated with the changes seen in the wild type wing discs in regions of endogenous Wingless signalling. While some folding of the epithelium and packing of cells occurred in response to Wingless, E-Cadherin (Figure 6A) and Armadillo (Figure 6B) expression were apparently unchanged at 0%. In contrast, in the subapical domain (10%), Wingless altered the subcellular distribution of E-Cadherin (Figure 6C) and Armadillo (Figure 6D). In particular, we observe increased colocalization of E-Cadherin and Armadillo puncta (Figure 6C and D). Interestingly, E-Cadherin seemed to be slightly depleted from the basolateral membrane whereas Armadillo was not, suggesting a change in the relationship of the two proteins upon Wingless stimulation.

In an effort to better document the changes in subcellular distribution of Armadillo and E-Cadherin seen upon Wingless signalling, we made use of an Armadillo construct that is constitutively active in the absence of ligand. Arm^S10^ lacks the Shaggy/GSK3 phosphorylation site (Figure 1) as well as the cactus-like residues for ubiquitination, both of which are required for protein degradation [30]. In addition to being Myc-tagged at the C-terminus, another useful property of this construct is the fact that the epitope for the N27A1 antibody is located within the deletion site (Figure 1), such that the endogenous Armadillo can be unequivocally distinguished from the exogenous Arm^S10^ [30].

**Figure 7:**
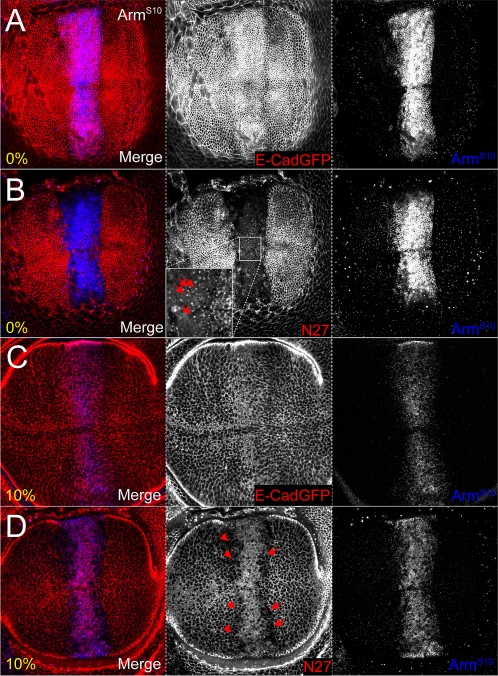
Arm^S10^ induced signalling correlates with clearing of endogenous Armadillo from the AJ and dynamic regulation of subcellular distribution subapically. (A) Similarly to Wingless ligand-dependent signalling, Arm^S10^ does not affect the distribution of E-Cadherin-GFP (E-CadGFP) in the AJ. (B) In contrast, Arm^S10^ entirely displaces endogenous Armadillo from the AJ, which can often be identified as puncta immediately subapically (red channel, inset). (C) E-Cadherin-GFP (E-CadGFP) accumulates in puncta upon Arm^S10^ overpexression similarly to ligand-dependent signalling. (D) In contrast, Arm^S10^ causes an accumulation of endogenous Armadillo to high levels in the centre of the *dppGAL4* overexpression domain, but results in complete loss from the cytoplasm and basolateral membrane at the edges (red arrows).

As with Wingless signalling, little change was seen in apicobasal polarity markers at the adherens and septate junctions, with E-Cadherin expression at 0% identical in Arm^S10^ and Wg overexpression experiments (compare Figures 6A and 7A; and data not shown). In contrast, Arm^S10^ was found to be more stable than endogenous Armadillo in the AJs (Figure 7B), the latter being completely excluded except for a punctate distribution immediately basal to the AJ (Figure 7B, red channel, inset).

More basally (10–50%; 10% shown), the distributions of apicobasal polarity markers did not differ appreciably upon overexpression of Arm^S10^ compared to Wingless (not shown). Most importantly, E-Cadherin retained the punctate accumulation seen upon Wingless signalling (Figure 7C, red channel) as well as the apparent weak basolateral membrane depletion within the lateral domain of expression. Thus overexpression of Wingless and Arm^S10^ appear to cause similar changes in E-Cadherin, at least at the subcellular level. In contrast, endogenous Armadillo showed a striking redistribution, with membrane, nuclear and punctate accumulation within the central domain of Arm^S10^ overexpression, and complete exclusion from the basolateral membranes laterally (Figure 7D red channel, arrows). Arm^S10^ was similarly distributed but lacked the dramatic modulation within the lateral expression domain. These data suggested that signalling might alter the subcellular distribution of Armadillo specifically at the membranes, with associated effects on the punctate species. Further, signalling appears to be correlated with the alteration of the physical association between Armadillo and E-Cadherin.

### Armadillo mutants reveal complex behaviour of endogenous Armadillo

The observation that Arm^S10^ modulates endogenous Armadillo at the AJ, in addition to its subcellular localisation and levels more basally, prompted us to study the effects of other Armadillo mutant proteins. These mutants should be able to associate with E-Cadherin through the Armadillo repeats, but are known to differ in their signalling ability (Figure 1). In particular, we were interested in how known deletion mutants would affect changes in the subcellular distribution of endogenous protein at the level of the AJs and basolateral membranes. The ΔNArm^1–128^, ΔNArm^1–155^ and Myr-ΔNArm^1–155^ mutants lack all regulatory motifs N-terminal to the Armadillo repeats, thus providing them with increased stability and activity. In addition, ΔNArm^1–155^ and Myr-ΔNArm^1–155^ are compromised in their ability to bind α-catenin, with Myr-ΔNArm^1–155^ differing in the presence of a Myristoyl tag targeting it to membranes. Finally, we compared these to the effect of mutant ArmΔC^XM19^ on Armadillo's subcellular distribution and signalling potential, as the C-terminus is known to act as a Wg transactivation domain (summarised in Figure 1).

**Figure 8:**
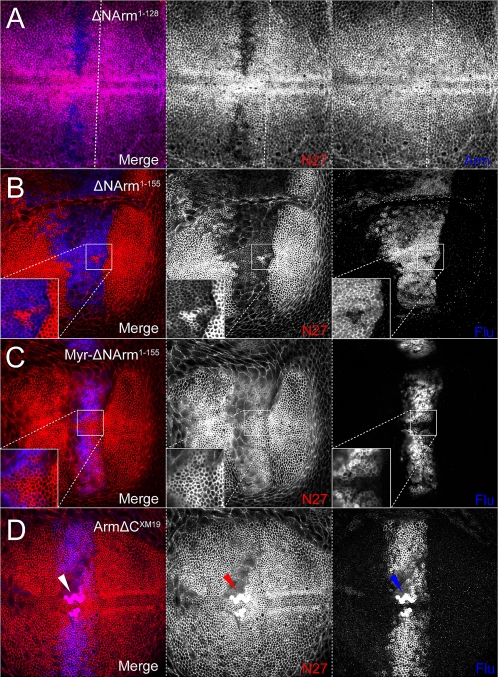
Stability of UAS constructs determines the strength of association of endogenous protein with the AJ. (A) ΔNArm^1–128^ excludes endogenous Armadillo (red channel, absence of staining) only at the strongest levels of *dppGAL4* expression, away from the A/P boundary (denoted by white line). (B) As with Arm^S10^, ΔNArm^1–155^ competes with endogenous Armadillo at the AJ (AJ) within the entire domain of *dppGAL4* expression, abutting the A/P boundary. However, some endogenous Armadillo remains (red, inset), and the construct is more diffusely associated with the AJ (blue, inset). The construct is also more diffusible, as demonstrated by the extent of its spread into more anterior AJs. (C) The myriostylation signal prevents Myr-ΔNArm^1–155^ from effectively binding in the AJ, allowing endogenous Armadillo to accumulate (red, inset), although it is strongly expressed both apically and basally in the membrane (blue, inset). (D) The C-terminal deletion ArmΔC^XM19^ is stable in the AJ, likely entirely excluding the endogenous protein. This is not verifiable with the available antibodies which detect both the construct and the endogenous Armadillo. Unlike in the other experiments, the protein(s) also accumulate at the D/V boundary.

At 0%, variation in the ability of the Armadillo mutants to associate stably with the AJ was observed, differing from Arm^S10^ in both strength and extent. Although untagged, ΔNArm^1–128^ can be distinguished from endogenous Armadillo by staining with both the “N27” (N-terminal, N27A1) and the “Arm” (central Armadillo repeats) antibodies, as the latter will identify both endogenous and overexpressed proteins (Figure 1). Compared to Arm^S10^, ΔNArm^1–128^ was found to be stable only in the central domain of *dppGAL4* expression, several cell diameters from the A/P boundary (Figure 8), where it inefficiently excluded the endogenous protein. In contrast, the Flu-tagged ΔNArm^1–155^ construct, which also lacks part of the 1^st^ Armadillo repeat (Figure 1), was found to be highly stable across the entire domain of *dppGAL4* expression (central and lateral), competing with the endogenous Armadillo up to the A/P boundary (Figure 8B), but not entirely excluding it from the AJ (compare insets red and blue channels), contrasting with the efficient exclusion caused by Arm^S10^. However, its distribution extended further anteriorly, particularly in the dorsal compartment (compare to Figure 7A and B). In contrast, the membrane targeted form Myr-ΔNArm^1–155^ was expressed in a similar domain as Arm^S10^, but was unable to exclude the endogenous protein from the AJ (Figure 8C, insets). Finally, the C-terminal-deleted form ArmΔC^XM19^ localised to the AJ similarly to Arm^S10^ (Figure 7D), probably competing effectively with the endogenous Armadillo protein. However, since neither the αN27 nor the αArm antibodies can distinguish this construct from endogenous protein, it was not possible to confirm this. Interestingly, this construct must be stabilised by Wingless as it was ectopically expressed apically near the D/V boundary (Figure 8D, arrowhead).

**Figure 9:**
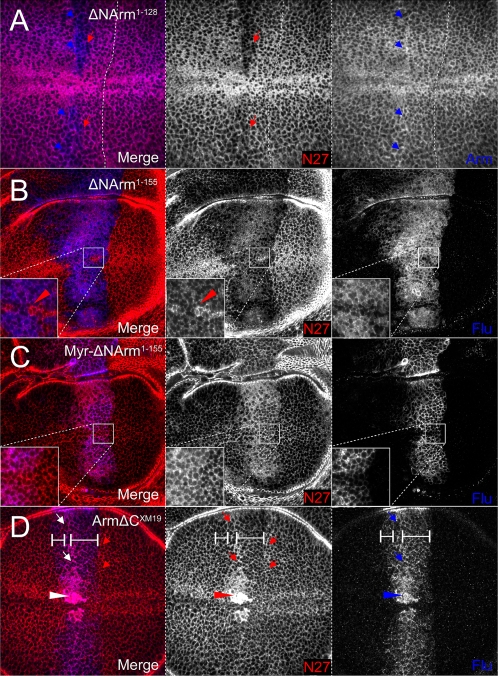
Strength of association of Armadillo with the basolateral membrane is dependent upon both the N- and C-termini. (A) UASΔNArm^1–128^ is targeted normally to the membrane, including the anterior “stripe” (blue arrows). Endogenous Armadillo is excluded (red arrows), but only in the domain of highest *dppGAL4* expression. (B) The ΔNArm^1–155^ construct is similar to Arm^S10^ but more soluble (red channel), excluding endogenous Armadillo from the membrane at the edges of the *dppGAL4* expression domain. However, few puncta are observed (red, inset). (C) The membrane-tethered Myr-ΔNArm^1–155^ is not freely diffusible (blue channel, inset) and drives endogenous Armadillo into the nucleus, but does not appear to alter its ability to associate with the membrane (red inset). (D) ArmΔC^XM19^, though diffusely associated with the membrane (blue), replaces endogenous Armadillo in the entire domain of expression (red channel, white scale bars), but is targeted to the anterior stripe like the endogenous form under wild type conditions (small arrows, all channels). ArmΔC^XM19^ is also weakly nuclear in Wg-receiving cells (arrowheads, all channels). Although the N27A1 antibody recognises both the construct and endogenous Armadillo, the endogenous protein also appears to localise more strongly in the nuclei (red channel).

More basally (10–25%), the mutant Armadillo proteins induced a combination of effects on endogenous Armadillo levels and subcellular localisation that represent a subset of those seen with overexpression of Arm^S10^. In particular, ΔNArm^1–128^ caused exclusion of endogenous Armadillo from the membrane (Figure 9A, red channel, red arrowheads). It also appeared to replace the endogenous protein in the anterior A/P stripe, immediately anterior to the central *dpp* expression domain (blue channel, blue arrowheads). ΔNArm^1–155^ overexpression obliterated this expression pattern, but induced a similar loss of Armadillo from the basolateral membrane, particularly in the lateral domains (Figure 9B). This appears to be caused by its own localisation, albeit patchily, in the membrane, and there is some evidence of its import into the nucleus (inset, blue channel). Exclusively targeted to the membrane (Figure 9C, blue channel), Myr-ΔNArm^1–155^ produced a clear nuclear accumulation of endogenous protein, with membrane-associated puncta being apparent (Figure 9C, red channel, inset). In stark contrast to the other mutants, ArmΔC^XM19^ was most stable at the D/V boundary where a fraction localised to the nucleus (Figure 9D, blue channel arrowhead), suggesting that it is responsive to Wingless signalling. Unlike the N-terminal deletion constructs (possibly with the exception of ΔNArm^1–128^), ArmΔC^XM19^ also clearly accumulated in the anterior A/P stripe more than elsewhere in the overexpression domain (arrows). Outside this stripe, it was apparently able to exclude the endogenous protein from the basolateral membrane, since αN27 staining was lower there than in surrounding wild type cells (Figure 9D, red channel, scale bars). This likely suggests that the endogenous protein was completely absent, and that the antibody was only detecting the ArmΔC^XM19^ protein. In conjunction, the data from the AJ and more basal sections suggest that the mutants cause a subcellular reshuffling of endogenous protein, possibly linked with its signalling ability.

### Non correlative distributions of Armadillo and E-Cadherin

The wild type distributions of E-Cadherin and Armadillo are tightly associated both at AJs and basolateral membranes, as well as in stripes at the anterior and posterior edges of a central domain, and in membrane-associated puncta (this study). We have shown that the Armadillo mutants caused dramatic changes in the subcellular distribution of endogenous Armadillo. In particular, N-terminal deletion constructs were effectively able to compete with endogenous Armadillo at the AJ, with the exception of Myr-ΔNArm^1–155^ which was targeted to all other membranes. Exclusion of Armadillo from membranes was correlated with the appearance of puncta as well as some nuclear accumulation. However, only ΔCArm^XM19^ was subject to regulation by Wingless at the D/V boundary. Since Wingless and Arm^S10^ signalling caused an apparent dissociation of E-Cadherin and Armadillo, we wanted to examine how these Armadillo mutants affected the distribution of E-Cadherin.

**Figure 10:**
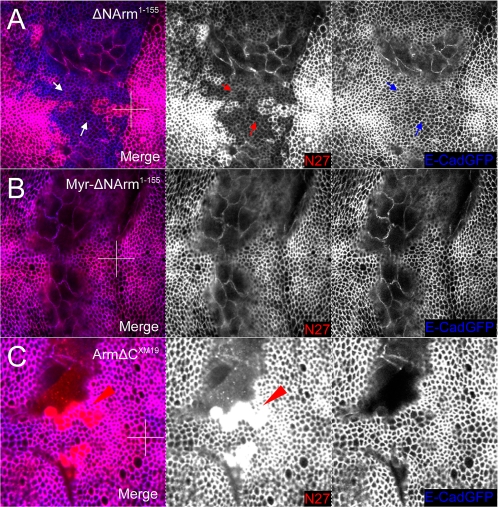
E-Cadherin levels change in the AJ in response to changes in levels of endogenous Armadillo protein upon N-terminal deletion mutant overexpression. (A) Overexpression of the ΔNArm^1–155^ construct with *dppGAL4* causes a reduction in E-Cadherin levels concomitant with a decrease in endogenous Armadillo (blue arrows). (B) No change is evident in E-Cadherin levels when the membrane tethered Myr-ΔNArm^1–155^ form is overexpressed. (C) Although levels of endogenous Armadillo increase at the level of the AJ upon overexpression of ArmΔC^XM19^ (red arrows), there is no change in levels of E-Cadherin relative to the wild type (blue arrowheads).

**Figure 11:**
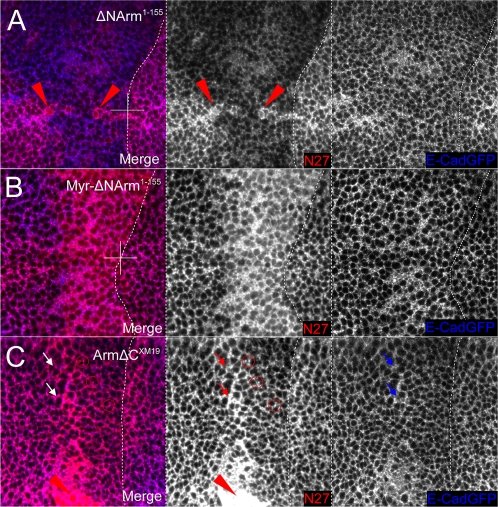
E-Cadherin levels change in the cytoplasm or basolateral membrane in response to changes in levels of endogenous Armadillo protein upon N-terminal deletion mutant overexpression. (A) E-Cadherin levels appear reduced in the domain of expression where endogenous Armadillo is excluded. Fewer puncta are also apparent relative to neighbouring wild type tissue upon overexpression of the ΔNArm^1–155^. (B) No change is evident in E-Cadherin levels when the membrane tethered Myr-ΔNArm^1–155^ form is overexpressed. (C) E-Cadherin levels are reduced in the domain of ArmΔC^XM19^ expression, and puncta are lacking. This is accompanied by an increase in the number of N27-postive puncta (red circles), representing endogenous or C-terminally truncated forms of Armadillo. There is also less E-cadherin associated with the anterior stripe, where endogenous Armadillo and E-cadherin perfectly colocalise under wild type conditions (red arrows). Note that in the wing disc in (C) only the dorsal aspect is shown, while (A) and (B) are show the intersection of the A/P and D/V boundaries.

At the AJ, E-Cadherin levels appeared to be somewhat reduced when overexpressing ΔNArm^1–155^ (Figure 10A, blue channel). This was in contrast to ΔNArm^1–128^ (not shown), Myr-ΔNArm^1–155^ and ΔNArm^XM19^, which like Arm^S10^, did not appear to affect E-Cadherin (Figure 10B, C). E-Cadherin levels at the basolateral membrane, or possibly in the cytoplasm, (10%) were reduced when overexpressing ΔNArm^1–155^, particularly at the D/V boundary where the *dppGAL4* domain intersects the Wingless signalling domain (Figure 11A, blue arrows). This slight reduction was not evident with Myr-ΔNArm^1–155^, where levels of E-Cadherin were either unchanged or slightly elevated (Figure 11B). In contrast, the ArmΔC^XM19^ construct caused a reduction of membrane E-Cadherin paralleling that of endogenous Armadillo where overexpressed, except at the anterior A/P stripe, where levels of ArmΔC^XM19^, and possibly endogenous Armadillo protein, appeared to be higher (Figure 11C). In addition, many Armadillo and E-Cadherin puncta were dissociated (red channel, circles). In combination, the discrepancy between the data from the AJ and the basolateral membrane suggested that the N- and C-termini play an important role in regulating E-Cadherin/Armadillo complex formation and subcellular distribution. Alternatively, association of the complex with other proteins might affect targeting of E-Cadherin and/or Armadillo to the membrane.

### A novel method to quantify changes in subcellular distribution of Armadillo and E-Cadherin

The observation that Armadillo mutants caused often subtle changes in the subcellular distribution of Armadillo and E-Cadherin suggested that a quantitative analysis might yield additional insight, and more importantly, allow an unbiased comparison across experiments. We therefore developed a method to quantify relative pixel intensity within and outside the *dppGAL4* domain in multiple sections and wing discs (see Materials and Methods for details). Our quantitative method required some internal control of endogenous expression to give an accurate measure of levels. This was not a concern with endogenous Armadillo or E-Cadherin, as wild type cells outside the overexpression domain act as the standard. However, in order to reliably compare mutant protein levels across experiments, it was necessary to set levels to equal 1 within the central or “high” domain of expression. This method allowed an estimate of relative levels of construct in the “low” expression or “lateral” domain relative to the “high” domain, as it considered these to be a proportion of 1. Thus the method allows a measure of construct stability, as the least stable constructs will be absent from the lateral domains of expression in which *dppGAL4* expression is lower. We therefore discuss the changes at different levels separately.

#### The adherens junction (AJ)

**Figure 12:**
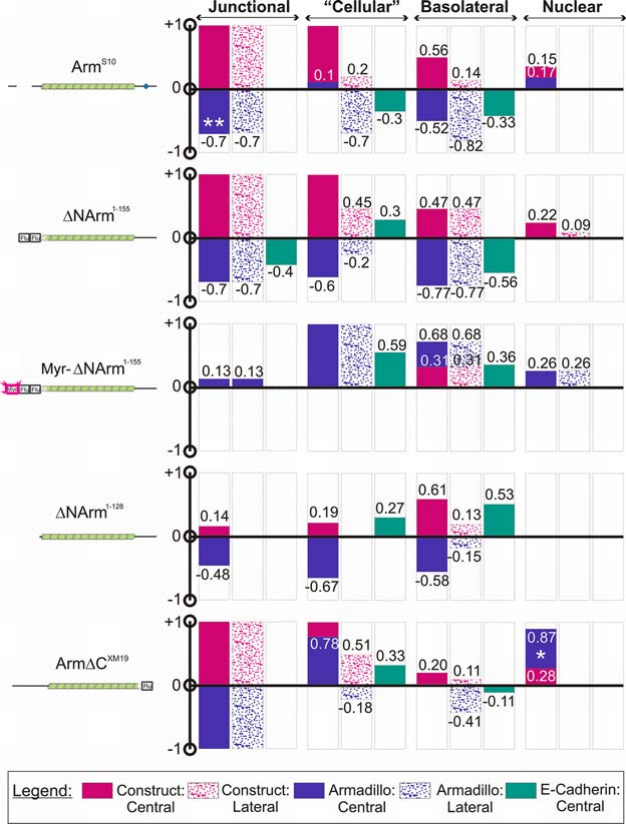
Summary of quantification of changes in levels upon Armadillo mutant overexpression. Levels of endogenous Armadillo (blue), overexpressed mutant (pink) and E-Cadherin (green) were assessed in (adherens) junctional, “cellular”, basolateral (membrane) and nuclear compartments. The “cellular” compartment assesses levels across a field of cells encompassing the basolateral membrane, nucleus and cytoplasm. Mutant constructs that were assessed are illustrated to the left of the graphs, and included Arm^S10^, ΔNArm^1–128^, ΔNArm^1–155^, Myr-ΔNArm^1–155^ and ArmΔC^XM19^. +1 and −1 represent maximal proportion changes in levels above and below the wild type baseline value of 0. Empty boxes indicate no change from wild type. *dppGAL4* driver levels are lower in “lateral” relative to “central” domains of expression, as reflected by spotted versus solid colours. E-Cadherin levels were not assessed in the nuclear compartment (N/A).

Comparing the levels of mutant protein in lateral relative to central domains, it was apparent that Arm^S10^, ArmΔC^XM19^ and ΔNArm^1–155^ were highly stable in the AJ, since equal proportions of mutant protein are seen in both domains (Figure 12). Similarly, although untagged, ΔNArm^1–128^ filled the junctions, likely to the exclusion of endogenous Armadillo (see below) but only in the central domain. This was apparent because the αArm antibody will detect both the construct and the endogenous Armadillo, and was 14% higher within the expression domain than without. In contrast, Myr-ΔNArm^1–155^ was not stable in AJs but rather was highly expressed in the basolateral membrane apically and basally, suggesting that the Myristoylation tag overcomes signals targeting it to the AJ.

The endogenous Armadillo protein levels tended to be inversely related to those of the overexpressed construct, suggesting that the endogenous form was excluded from the AJ. Quantification revealed that endogenous Armadillo levels in the AJ decreased by 70% in the case of Arm^S10^ and ΔNArm^1–155^ and by almost 50% in the case of ΔNArm^1–128^, but the latter only in the central domain of expression (Figure 12). Overexpression of Arm^S10^ in fact caused an almost total loss of Armadillo at the AJ. The underestimate by the quantification method was caused by the presence of puncta immediately basal to the AJ (Figure 7B; compare with Figure 8B), which inflated the pixel intensity values measured. Since all three N-terminal deletion mutants produce similar effects at the AJ, it is likely due to their increased stability relative to the endogenous form, mediated in large part by their escape from Shaggy/GSK3 regulation.

The situation with ArmΔC^XM19^ and Myr-ΔNArm^1–155^ was somewhat different to the other mutants. Myr-ΔNArm^1–155^, which is targeted to all membranes but not the AJ, appeared to produce a slight increase in Armadillo at the AJ compared to wild type (+13%, Figure 12). This was similar to that caused by overexpression of ΔNArm^1–128^ in the central domain (+14% Figure 12). These data perhaps indicate that, unless Armadillo can move freely between the membrane and the AJ, its expression becomes “patchy” at the AJ. In contrast, ArmΔC^XM19^ filled the AJ; however, since αN27 detects both the construct and endogenous Armadillo, it is impossible to determine how the endogenous protein levels change. Nevertheless, given the stability of ArmΔC^XM19^ in both the central and lateral domains of expression, it is likely that much of the endogenous protein was excluded. Taken together, these data highlight a complex and dynamic relationship between different subcellular compartments of Armadillo.

In contrast to changes in levels of Armadillo induced by the different mutant forms, quantification supported the observation that E-Cadherin levels change little in the AJ upon mutant overexpression (changes of less than 10% are not shown), with the exception of ΔNArm^1–155^, which caused a 40% decrease in E-Cadherin levels (Figure 12). These data underscore the fact that the majority of mutants did not strongly affect traffic of E-Cadherin to the AJs, in spite of changes to endogenous Armadillo levels there.

#### “Cellular” levels

The “cellular” compartment, as defined here, includes cytoplasm, nuclear and basolateral membrane components. In order to estimate levels of mutant overexpression, which cannot be compared to wild type cells as for Armadillo and E-Cadherin, we defined “lateral” and “central” domains of overexpression, reflecting strength of the *dppGAL4* driver expression (high centrally, lower laterally, see Materials and Methods). This further provides an estimate of “stability” since only those proteins that are highly stable will continue to be expressed in lateral domains. Employing this rationale to evaluate different mutants, we found that both ΔNArm^1–155^ and ArmΔC^XM19^ levels in the lateral domains were approximately 50% of the maximal levels seen in the central domain (0.45 and 0.5, respectively, Figure 12). In contrast, Arm^S10^ only represented 20% of maximal levels, and Myr-ΔNArm^1–155^ (as a true membrane-marker) was absent from the cytoplasm. αArm, which detects both ΔNArm^1–128^ and endogenous protein, showed an increase of 19% of the pixel intensity in the central domain relative to the surrounding wild type tissue, indicating that the construct was not very stable in the “cellular” domain. This was further shown by the absence of difference between Armadillo levels in the lateral domain of expression compared to the wild type (Figure 12).

Interestingly, the mutants differed dramatically in the magnitude and direction of effects on levels of endogenous Armadillo in the “cellular” domain, highlighting a qualitative difference in lateral and central domains of expression. For instance, in the central domain both ΔNArm^1–155^ and ΔNArm^1–128^ caused a dramatic reduction in endogenous Armadillo levels, with decreases of 60 and 67% respectively (Figure 12). In contrast, Myr-ΔNArm^1–155^ and ArmΔC^XM19^ caused a twofold and 78% increase, respectively, in cellular Armadillo levels, whereas only a 10% increase was apparent upon Arm^S10^ overexpression. Thus, in the central domain where overexpression levels are highest, two classes of mutant can be distinguished: those that radically increase, and those that decrease Armadillo levels, respectively. Furthermore, these changes do not necessarily correspond to those seen in the AJ.

In lateral domains, where mutant overexpression levels are lower, only Myr-ΔNArm^1–155^ showed similarly high levels of endogenous protein as in the central domain (Figure 12). At the opposite end of the spectrum, ΔNArm^1–128^ had no effect at all, such that Armadillo levels in the lateral domain were equal to those in neighbouring wild type cells. However, ΔNArm^1–155^ and ArmΔC^XM19^ here caused almost 20% reduction in endogenous levels; interestingly the levels of mutant expression also closely correspond (near 50%). Arm^S10^ caused the greatest reduction in Armadillo protein in the lateral domain of expression. The absence of obvious correlation between levels in lateral and central domains highlights qualitative differences in the mutants.

Unlike the construct and endogenous Armadillo levels, which at least partially paralleled one another, changes in cellular E-Cadherin levels were much less variable in direction (Figure 12). In effect, levels increased in all cases except Arm^S10^, which caused a 30% decrease in E-Cadherin levels. ArmΔC^XM19^, ΔNArm^1–128^ and ΔNArm^1–155^ promoted increased E-Cadherin levels approaching 30% within the (central) domain of expression, and Myr-ΔNArm^1–155^ by almost 60%. These observations were intriguing given that only ΔNArm^1–155^ caused appreciable changes in E-Cadherin levels at the AJ, suggesting that we were missing some level of complexity in our evaluation.

Since the quantification method used estimated changes in protein levels across a field of cells, we further subdivided the “cellular” domain into basolateral membrane and nuclear components, by specifically choosing and quantifying points within these compartments. The expectation was that comparison of the total “cellular” levels with those in its constituent parts would allow a more accurate estimate of the subcellular location at which changes in Armadillo and E-Cadherin were occurring.

#### Basolateral membrane levels

Using discs that were not saturated for fluorescence intensity, and that had approximate 1∶1 ratio of endogenous Armadillo to E-Cadherin proteins in wild type cells outside the overexpression domain, nevertheless does not provide a very accurate means of assessing the construct levels across experiments, even when attempting to normalise to the maximal pixel intensity. Thus maximal expression levels in the central domain of 0.56, 0.47, 0.68 and 0.2 for Arm^S10^, ΔNArm^1–155^, Myr-ΔNArm^1–155^ and ArmΔC^XM19^ mutants, respectively, only provided a rough estimate. What was apparent, however, was that ΔNArm^1–155^ and Myr-ΔNArm^1–155^ were as stable in the lateral as in the central domains of expression. This was in contrast to the other constructs, which were either only stable in the highest domain of *dpp* expression (ΔNArm^1–128^), or at much lower levels (Arm^S10^, 14% and ArmΔC^XM19^, 11%, Figure 12).

Levels of endogenous Armadillo in the basolateral membrane tended to parallel those within the “cellular” compartment in direction if not magnitude. This was true of ΔNArm^1–155^, Myr-ΔNArm^1–155^ and ΔNArm^1–128^, and of Arm^S10^ in the lateral domain. In the central domain, Arm^S10^ caused a dramatic reduction of endogenous Armadillo (−52%) while “cellular” levels remained relatively unchanged (+10%), suggesting a change in cytoplasmic or nuclear protein levels.

In the absence of nuclear E-Cadherin, the basolateral membrane E-Cadherin should in principle constitute the entire “cellular” component, assuming there was no cytoplasmic protein. The E-Cadherin puncta observed in the wing discs likely represent the cytoplasmic component. Arm^S10^, ΔNArm^1–128^ and Myr-ΔNArm^1–155^ basolateral membrane levels were similar in direction to the quantified “cellular” levels. Arm^S10^ showed similar decreases of E-Cadherin levels both across the cellular field and in the basolateral membrane (0.3 and 0.33). Myr-ΔNArm^1–155^ and ΔNArm^1–128^ caused increases in basolateral membrane-associate E-Cadherin relative to wild type sister cells (Figure 12). Most importantly, ΔNArm^1–155^ showed a change in direction of E-Cadherin levels between the “cellular” compartment and the basolateral membrane. This clearly indicates a movement of E-Cadherin from the basolateral membrane to the cytoplasm or to the nucleus, and may correlate with the loss of endogenous Armadillo there (Figure 11A and 12).

#### Nuclear levels

One of the most intractable problems faced in immunohistochemical studies of Armadillo concerns the difficulty of unequivocally showing that it accumulates to any appreciable levels in the nucleus upon signalling. This is particularly relevant in cases where it is important to distinguish low levels from background “noise”. We therefore further extended our quantification methodology to help address any small changes in nuclear Armadillo accumulation that might have occurred upon mutant overexpression, by comparing levels within the expression domain with those in wild type neighbours. Since the nucleus constitutes the largest fraction of the cells in the wing imaginal disc epithelium, it is relatively straightforward to assign levels there.

As with the basolateral membrane quantification, nuclear levels were calculated relative to a maximal pixel intensity of 255, after taking into consideration “background” pixel intensity. Construct expression in nuclei was rarely high if observable, with Myr-ΔNArm^1–155^ and ΔNArm^1–128^ mutants undetectable in the nucleus. Only ΔNArm^1–155^ and Arm^S10^ had appreciable localisation in the nucleus in the central domain of *dppGAL4* expression, represented as 0.22 and 0.15 of a possible maximum of 1. ΔNArm^1–155^ was also present at low levels in the lateral domain of expression, with a value less than 10% (0.09, Figure 12). ArmΔC^XM19^ was only present in nuclei in cells adjacent to the source of Wingless (0.28, white star), at the D/V boundary.

Endogenous Armadillo was also found in nuclei, paralleling Arm^S10^ in localisation and levels (Figure 12). In contrast, ΔNArm^1–155^ did not cause a visible translocation of Armadillo to the nucleus, similarly to ΔNArm^1–128^. The membrane localisation of Myr-ΔNArm^1–155^, however, induced a uniform increase in nuclear Armadillo levels throughout the *dppGAL4* expression domain. Finally, overexpression of ArmΔC^XM19^ appeared to induce some increase in endogenous protein levels in the nucleus, as there was more αN27 staining in the nuclei than could be accounted for by ArmΔC^XM19^ alone (Figure 12, white star), even when pixel intensity on the red channel was adjusted to equal that of the blue channel (*i.e.* if all N27 levels corresponded to those of ArmΔC^XM19^; data not shown). The data presented here highlight the value of quantifying the changes in subcellular distribution of Armadillo and E-Cadherin, which otherwise might not be visible to the naked eye.

### Changes in Armadillo distribution and levels correlate with adult phenotype

Wingless signalling, in combination with Notch, is responsible for the formation of bristles along the wing margin through its role in the positioning and stabilisation of sensory organ precursors on either side of the D/V boundary [55], [56]. By extension, the stabilisation of Armadillo can induce the formation of ectopic bristles both at the margin and within the wing blade. As such, the presence or absence of ectopic bristles acts as a readout of Wingless signalling and Armadillo stability in the wing. Since there is evidence that the levels of Armadillo alone might not be a signalling determinant, we wanted to examine the phenotypes caused by overexpression of Armadillo mutant proteins, and to attempt to correlate them with the changes in subcellular distribution and levels that we observed.

**Figure 13:**
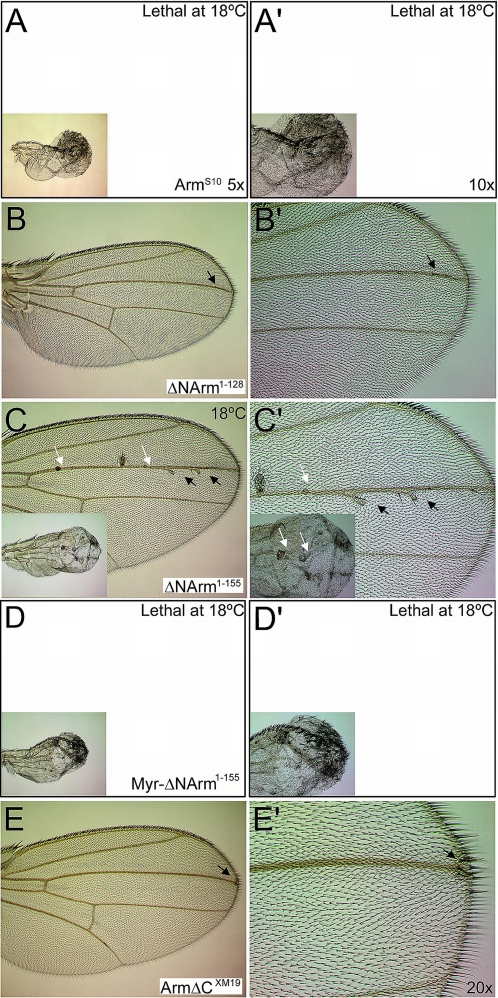
Armadillo mutants overexpressed with *dppGAL4* induce ectopic bristles and veins in adult wings, with varying degrees of severity. (A, A′) Arm^S10^ is lethal even at 18°C using *dppGAL4*, but results in a lawn of ectopic bristles using *C5GAL4* (insets). (B, B′) Only a very weak phenotype is caused by overexpression of ΔNArm^1–128^, inducing ectopic bristles on vein L3 near the wing margin (arrowheads). (C, C′) Although ectopic bristles and veins are induced at 18°C (arrowheads), the phenotype caused by ΔNArm^1–155^ is predominantly neurogenic, with many ectopic sensillae along the wing veins (white arrows and inset, *C5GAL4*). (D, D′) In contrast, even at 18°C no pupae eclose when the tethered form is overexpressed with *dppGAL4*. The phenotype induced by Myr-ΔNArm^1–155^ is similar to, though more severe than, that of Arm^S10^, causing a lawn of ectopic bristles to form with *C5GAL4* (inset). (E, E′) ArmΔC^XM19^ has a very weak phenotype, with only few ectopic bristles forming at the wing margin at the tip of vein L3 (arrowheads).

As with overexpression of Wingless under *dppGAL4*, overexpression of Arm^S10^ or Myr-ΔNArm^1–155^ was lethal even at 18°C (Figure 13A, A′, D, D′). A few wings, however, were recovered that overexpressed ΔNArm^1–155^ (Figure 13C, C′), exhibiting expanded sensillae (white arrows), as well as additional bristles associated with ectopic veins near the anterior extent of *dpp* expression. However, using *C5GAL4*, which drives late expression throughout the wing pouch with the exception of the D/V boundary, it was possible to compare phenotypes across these constructs. Although the wings were severely folded and blistered, it was evident that while ΔNArm^1–155^ caused a neurogenic phenotype, the ectopic signalling induced by Arm^S10^ and Myr-ΔNArm^1–155^ resulted in a lawn of ectopic bristles (Figure 13A, A′, C, C′, D, D′, insets). In contrast, only weak phenotypes were observed with ΔNArm^1–128^ and ArmΔC^XM19^ wings, with few ectopic bristles along and at the tip of the L3 vein, respectively (Figure 13B, B′, E, E′). The presence of ectopic bristles correlated well with the high nuclear levels of endogenous Armadillo induced by overexpression of Arm^S10^, ΔNArm^1–155^ and Myr-ΔNArm^1–155^; with ArmΔC^XM19^ near the D/V boundary (Figure 12); and with nuclear localisation of αSenseless, which marks sensory organ precursors (not shown). However, the mainly neurogenic and veination defects of ΔNArm^1–155^, which differ both quantitatively and qualitatively from both the Myr-ΔNArm^1–155^ and ΔNArm^1–128^ phenotypes, suggested that the severity of the effects might lie at least partly in disruption of interactions between Armadillo and α-catenin. The ΔNArm^1–155^ and ΔNArm^1–128^ mutants differ structurally in the extent of the N-terminal deletion; the absence of part of the 1^st^ Armadillo repeat in ΔNArm^1–128^ likely reduces its binding to α-catenin (Figure 1).

### Mutants differentially affect the actin cytoskeleton at the A/P boundary

**Figure 14:**
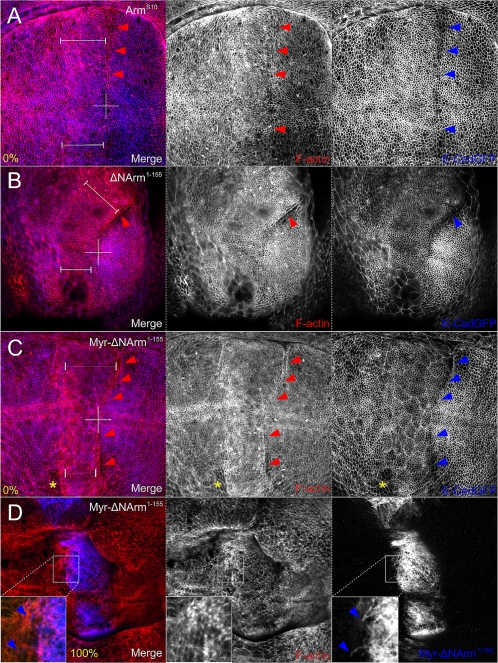
Phalloidin staining reveals a strong F-actin cable at the A/P boundary corresponding with reduced E-Cadherin in ΔNArm^1–155^ and Myr-ΔNArm^1–155^, but not in Arm^S10^. The extent of the overexpression domain is indicated with dimension lines, as assessed by staining (not shown here). (A) Although F-Actin staining clearly indicates the aligned cells at the A/P boundary with Arm^S10^, no change in levels was observed. (B) In contrast, overexpression of ΔNArm^1–155^ results in stretching of the A/P cells and an increase of F-Actin (red arrowhead), corresponding to a region of low E-Cadherin expression (blue arrowhead). Note the folding of the epithelium which reveals peripodial membrane cells. (C) Similarly, Myr-ΔNArm^1–155^ causes stretching and F-Actin accumulation at the A/P boundary where E-Cadherin is lower (red and blue arrowheads), and even appears to induce boundary cell-like behaviour at the anterior extent of mutant overexpression (yellow asterisk). (D) Basally, Myr-ΔNArm^1–155^ uniquely causes the formation of filopodia, the base of which are associated with F-Actin bundles (inset).

Recently, the dogma that α-catenin can simultaneously bind the E-Cadherin/β-catenin complex and the cytoskeleton has been challenged [49], [50]. Further, Major and Irvine [57] have reported that actin, in response to Notch signalling, is responsible for cell sorting and shape changes at the D/V boundary in wing imaginal discs. Our results indicating that Armadillo mutants differentially alter the subcellular distribution of endogenous Armadillo and E-Cadherin, in addition to the observation that cells align at the A/P boundary, and that the mutants likely differ in their ability to bind α-catenin (Figure 1), led us to examine the distribution of F-actin. We found that the changes in tension of cells along the A/P boundary upon overexpression of Arm^S10^, ΔNArm^1–155^ and Myr-ΔNArm^1–155^ were correlated with differing levels of actin accumulation both at the AJ (Figure 14), and basally (not shown). In particular, while Arm^S10^ produced little effect (Figure 14A), both ΔNArm^1–155^ and Myr-ΔNArm^1–155^ caused a strong accumulation of actin, as assessed by phalloidin staining, along the A/P boundary. What resembled an actin “cable” corresponded most strongly with reduced E-Cadherin levels in the boundary cells (Figure 14B, C). Further, this phenomenon was also observed at the anterior extent of the Myr-ΔNArm^1–155^ expression domain, as though cells were adopting a boundary-like fate there (Figure 14C, yellow asterisk).

Basally (100%), cells expressing Myr-ΔNArm^1–155^ often extended filopodia-like processes towards the anterior compartment, which were associated though not coincident with, actin foci (Figure 14D and insets). This was never observed with other mutants, and the filopodia were never seen to express either E-Cadherin or endogenous Armadillo (not shown). However, the observation that filopodia were also positive for the αArm antibody confirms that they did in fact contain the entire Myr-ΔNArm^1–155^ mutant protein (not shown). Taken together, these data suggest that the mutant Armadillo proteins act trough a combination of changes to the relationship between endogenous Armadillo, E-Cadherin, and the actin cytoskeleton.

## Discussion

We present here a detailed *in vivo* analysis of the subcellular distribution of Armadillo, the *Drosophila* orthologue of ß-catenin, in third instar larval wing discs, with a particular focus on the Armadillo located at the AJ. We show that the pattern of Armadillo undergoes dynamic spatial and temporal regulation in response to Wingless signalling. Significantly, our observations suggest that this event leads to the dissociation of Armadillo and E-Cadherin protein localisation, and accumulation of punctate Armadillo in a subapical compartment.

It has been broadly assumed that the key component of Wnt signalling is the stabilisation and subsequent rise in concentration of a cytoplasmic pool of ß-catenin (reviewed in [1]). However, there is evidence that the concentration of β-catenin alone is not a determinant of Wnt signalling [35], [58]–[61], and it is becoming increasingly clear that other factors, like nuclear shuttling and cytoplasmic tethering, affect the nuclear availability of ß-catenin, and thus its ability to interact with the transcriptional machinery [39], [62]. It is possible that the association of ß-catenin with E-Cadherin also influences its activity under steady state conditions, not only in pathological or overexpression situations.

Altogether our observations indicate an important relationship between the distribution and signalling activity of Armadillo, rather than simply its levels. The independence of signalling activity on Armadillo levels has been previously demonstrated in a variety of contexts [35], [58], [59]. We confirm and extend these conclusions using a novel quantification method that allows estimation of relative Armadillo levels in specific subcellular compartments of the cell.

Several lines of evidence hint at the existence of a subapical compartment linking endosomal recycling pathways to signalling, to which E-Cadherin and ß-catenin might be targeted during AJ remodelling [63], [64]. Our *in vivo* data indicating that Wg signalling is correlated with the appearance of Armadillo puncta in a subapical domain, both under wild type and experimental conditions, represents circumstantial support for the existence of a subapical signalling domain. However, the nature of the punctate species of Armadillo remains to be elucidated. In this regard, the dissociation that we observe between E-Cadherin and Armadillo distributions upon overexpression of Wingless or Arm^S10^ was intriguing. In a variety of systems, overexpression of E-Cadherin effectively blocks Wnt signalling by sequestering or titrating available ß-catenin at the membrane [31], [40], [65]. This titration has been thought to take place from a cytoplasmic pool. However, it is also possible that Armadillo is released for signalling from a membrane-associated pool. In this case, E-Cadherin would act as an anchor for a signalling pool of Armadillo rather than sequestering the cytoplasmic pool. While much evidence suggests that adhesion and signalling are mutually exclusive states, it is more likely that these in fact represent alternate faces of the same coin, determined in large part by a balance of tyrosine kinase and phosphatase activities of the E-Cadherin/catenin complex [1].

E-Cadherin plays a central role in epithelial-mesenchymal transitions associated with cancers, through deregulation of appropriate ß-catenin function ([15] and reviewed in [66];). Our data on Armadillo mutants further point to an active role of Armadillo in determining the subcellular localisation of E-Cadherin, and hence function in adhesion or Wg signalling. In this context it is perhaps noteworthy that ΔNArm^1–155^, the mutant with the most significant effect on levels and localisation of E-Cadherin/Armadillo at the AJ and basolateral membrane, also appeared to cause sorting defects in wings (refer to Figure 13C). In principle all mutant proteins used here should be able to bind E-Cadherin through the Armadillo repeats [67], [68], their stability and activity dependent upon mutation of residues in the N-terminus rather than an inefficiency of binding to E-Cadherin [30], [35], [69].

Some of the effects that we observe in larval wing discs may be attributed to titration of regulatory factors by the mutant forms of Armadillo, as has been reported elsewhere [4], [34]. In particular, all overexpressed mutants may sequester Axin and APC, thereby relieving the negative regulation of endogenous Armadillo [39], [70]–[72]. Furthermore, Arm^S10^ is able to bind Legless and Pygopus to activate Wnt targets in the nucleus, an interaction unlikely to occur with ΔNArm^1–155^ which lacks part of the 1^st^ Armadillo repeat [73]. It is probable that ΔNArm^1–155^ titrates positive regulators in the nucleus, which could explain its phenotype in wings, which is neurogenic or adhesive, rather than reminiscent of ectopic signalling when compared with Arm^S10^ or Myr-ΔNArm^1–155^. Finally, we show that ArmΔC^XM19^ not only accumulates in membranes, but also in nuclei at the D/V boundary, where it is responsive to Wg. Previous reports suggested that the mutant allele *arm^XM19^*, which causes segment polarity and cuticle defects in embryos, retains some signalling potential [32]. The ability of ArmΔC^XM19^ to weakly activate the Wg pathway may be due to its inability to bind Chibby at the C-terminus, a negative regulator of Wnt signalling [34], [74].

We do not find a simple correlation between subcellular distribution and activity of the mutant forms of Armadillo that we used in our study. It has been reported in embryos that the signalling activity of Myr-ΔNArm^1–155^ is mediated exclusively through endogenous Armadillo, which is driven into nuclei to active Wg targets [34]. Our results are consistent with this, as we observed an increase in nuclear Armadillo throughout the Myr-ΔNArm^1–155^ overexpression domain. In contrast, in the case of ΔNArm^1–155^ we can detect some of the protein in the nucleus, similarly to Arm^S10^. However, although ΔNArm^1–155^ efficiently occupies basolateral membranes, Armadillo levels in the nucleus do not rise appreciably, except in a few cells at the D/V boundary next to the source of Wingless, suggesting that this protein is sensitive to some Wingless regulation that is not Sgg/GSK3-dependent. Another striking difference between these mutant proteins is that while Myr-ΔNArm^1–155^ leaves the AJ unaffected, ΔNArm^1–155^ reduces Armadillo/E-Cadherin levels. Taken together, these observations lead us to suggest that the basolateral membrane and AJ-associated pool of E-Cadherin/Armadillo are criticial to signalling.

**Figure 15:**
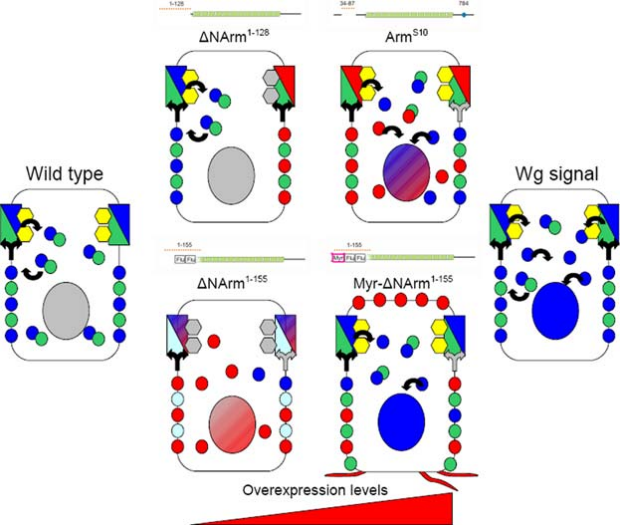
Theoretical model of the localization and interactions of wild type and mutant forms of Armadillo and E-Cadherin. In a wild type cell in its basal state (no signalling, left), Armadillo (blue dots) and E-Cadherin (green dots) are targeted to the membrane, possibly via the exocyst complex [43]. From there, Armadillo/E-Cadherin cycle to the Adherens Junction (AJ, box), or are hypothesised to remain in the cytoplasm as a complex free from degradation. There is little Armadillo in the nucleus (grey oval). During Wingless signalling (right), Armadillo accumulates in subapical puncta and can enter the nucleus (blue oval) to activate Wg targets. We hypothesise that Armadillo/E-Cadherin are released from the AJ (black arrows); alternatively the rate of cycling of Armadillo/E-Cadherin may also be increased. In our proposed model, either in its basal state or during signalling, Armadillo/E-Cadherin may be able to bind α-catenin (yellow hexagon). The activated N-terminal deletion mutants (centre, red boxes and dots) represent a range of effects in the spectrum between the basal state and Wingless signalling, depending at least in part on overexpression levels (bottom, red bar). ΔNArm^1–128^ (centre, top left) most closely resembles the basal state, with little Armadillo in the nucleus or signalling. At highest levels of overexpression, ΔNArm^1–128^ excludes Armadillo from the basolateral membrane and AJ, and is unlikely to efficiently bind α-catenin (grey hexagon; [76]). Arm^S10^ (centre, top right) excludes Armadillo from the AJ, as well as basolateral membranes at lowest expression levels. However, Arm^S10^ has no effect on E-Cadherin levels and should have the capacity to bind α-catenin [30], [76], thus ensuring appropriate adhesive function, as well as signalling (red and blue nucleus). In this case, we propose that Armadillo/E-Cadherin at the basolateral membrane may not be sufficiently stable to enter the AJ (grey arrow). Several sources indicate that Armadillo/E-Cadherin is first targeted to the basolateral membrane, and from there to the AJ [42], [78]. Myr-ΔNArm^1–155^, while unable to bind α-catenin [76], nevertheless ensures adhesive function through Armadillo/E-Cadherin at the AJ. These would be able to efficiently cycle between the subcellular compartments and enter the nucleus (blue oval). Additionally, filopodia extend into the environment from the basal side of cells expressing Myr-ΔNArm^1–155^. ΔNArm^1–155^, in contrast, impedes proper Armadillo/E-Cadherin function at the basolateral membrane and AJ through a reduction in their levels (fewer blue dots, pale green). It is unable to bind α-catenin, but enters the nucleus where it may interact with the transcriptional machinery. Note that all representations of cytoplasmic protein localisation are inferred from changes in the other more easily quantifiable compartments (e.g. “cellular” = nucleus+basolateral membrane+cytoplasm).

One of the possibilities raised by our study that might help to explain discrepancies in the signalling ability of our N-terminal deletion mutants is that some cell-surface protein is required for the proper shuttling of Armadillo/E-Cadherin among the subcellular compartments. A putative candidate might be α-catenin, whose passive role in linking the E-Cadherin/ß-catenin complex to the actin cytoskeleton has recently been put into question [49], [50]. Indirect evidence for a role of α-catenin in mediating the signalling or stability of Armadillo arises from several observations. First, Arm^S10^, which is functional in both signalling and adhesion, is likely the only activated form able to efficiently bind α-catenin, with perhaps the exception of ArmΔN^1–128^ [30], [75], [76]. Second, although Myr-ΔNArm^1–155^ lacks α-catenin binding sites, its targeting to the membrane may overcome this limitation by bringing it into proximity with cell surface molecules. Additionally, junctional function is not compromised as endogenous Armadillo and E-Cadherin occupy the AJ. ΔNArm^1–155^, on the other hand, can neither bind α-catenin nor is able to effectively recruit proteins near the cell surface, and furthermore impedes normal Armadillo/E-Cadherin function at the AJ. Finally, our data suggest that actin accumulates at the A/P boundary in cells depleted of E-Cadherin, most notably upon overexpression of ΔNArm^1–155^. We therefore see a correlation between signalling of mutants, either alone or through endogenous Armadillo, and their ability to interact with α-catenin. It is interesting in this context to note that α-catenin may inhibit CK1 phosphorylation-dependent degradation of β-catenin, and that the region encompassing the junction of the N-terminus and first Armadillo repeat are critical for this regulation [77].Thus a possible model of Armadillo/E-Cadherin movement upon signalling can be derived from our mutant data, from which we infer a regulatory input from α-catenin (Figure 15). In support of our model, plasma membrane recruitment of a punctate, signalling-competent species of ß-catenin appears to be an important step in the transcriptional activation of Wnt signalling both *in vitro* and *in vivo*, and occurs independently of E-Cadherin [81]. Additional studies will be required to confirm the validity of this model, as well as any putative role of α-catenin in mediating these interactions.

### Conclusions

We present here an analysis of the subcellular distribution, levels and activity of mutant and endogenous Armadillo in the wing imaginal disc. We find that there is no simple correlation between the amount of Armadillo and its activity. Additionally, the degree of signalling by the endogenous Armadillo is dependent on the activity of the activated form. More significantly, the subcellular localization of Armadillo may be critical to its function.

One of the most important implications of our data is that there is a connection between the Armadillo/E-Cadherin complex and Wingless signalling. One possibility is that the Armadillo that is involved in signalling is derived from the AJs, where it is tethered by E-Cadherin. Either Wingless signalling induces the release of this pool, or it changes the rate at which Armadillo/E-Cadherin cycle through different subcellular compartments. It remains to be tested whether or not α-catenin, along with the actin cytoskeleton, play a role in regulating this complex.
